# Exercise-Induced FNDC5/Irisin Ameliorates Cognitive Impairment in Aged Mice, Associated with Antioxidant and Neurotrophic Responses

**DOI:** 10.3390/antiox14101239

**Published:** 2025-10-15

**Authors:** Jae Min Lee, Tae Hyeok Sim, So Hee Kim, You Jung Choi, Joo Hee Lee, Seung Geun Yeo, Youn-Jung Kim

**Affiliations:** 1 College of Nursing Science, Kyung Hee University, Seoul 02447, Republic of Korea; jmlee3042@khu.ac.kr (J.M.L.); lovejjoo2@naver.com (J.H.L.); 2 Department of Otorhinolaryngology Head & Neck Surgery, College of Medicine, Kyung Hee University Medical Center, Seoul 02447, Republic of Korea; yeo2park@khu.ac.kr; 3 Department of Nursing, Graduate School, Kyung Hee University, Seoul 02447, Republic of Korea; xogur957@khu.ac.kr (T.H.S.); kprenurse@gmail.com (S.H.K.); choiyj9825@naver.com (Y.J.C.); 4 College of Nursing, Woosuk University, 443 Samnye-ro, Samnye-eup, Wanju_Gun, Wangju 55338, Republic of Korea

**Keywords:** aging, exercise, cognitive impairment, FNDC5/irisin, Nrf2

## Abstract

Aging contributes to neurodegeneration, predominantly characterized by increased oxidative stress, which leads to neurodegenerative changes and cognitive decline. This cognitive impairment is often associated with neuroinflammation, oxidative stress, and neuronal damage. Exercise is widely recognized for its capacity to elevate levels of irisin, a hormone derived from the cleavage of fibronectin type III domain-containing protein 5 (FNDC5). FNDC5/irisin acts as a myokine that mediates numerous beneficial effects of physical activity on metabolic health. It has also been recognized for its neuroprotective roles, suggesting its potential to mitigate neurodegenerative processes by promoting neuronal survival, reducing oxidative stress, and enhancing synaptic plasticity. However, the specific impact of exercise on the FNDC5/irisin pathway and antioxidant mechanisms in the aged brain remains insufficiently explored. In this study, we aimed to validate the neuroprotective role of exercise-induced FNDC5/irisin against aging-related oxidative stress, glial activation, neuronal damage, and cognitive impairment in 20-month-old mice. The exercise group underwent treadmill running for 60 min daily over an 8-week period. Our findings indicated that aging mice exhibited cognitive impairment, as evidenced by the Y-maze test; however, treadmill exercise effectively alleviated this impairment. Aged mice showed the activation of microglia and astrocytes in the hippocampus, which was notably reduced by exercise. Moreover, exercise improved the levels of calbindin and irisin, which were diminished due to aging. Our study demonstrated that aging led to a decrease in the antioxidant response element system and FNDC5/irisin pathway. However, exercise effectively activated Nrf2 and FNDC5/irisin expression, subsequently enhancing levels of SOD1, GSTO1/2, Sirt1, PGC-1α, BDNF, IGF-1, and IGF-2 in the hippocampus. The exercise-induced activation of Nrf2 signaling and FNDC5/irisin has emerged as a potent mechanism for alleviating oxidative stress and neuroinflammation associated with aging. In conclusion, our findings suggest that regular exercise has the potential to alleviate cognitive impairment through the activation of PGC-1α-FNDC5/irisin signaling, the Nrf2 ARE system, and neurotrophic factors in aged mice.

## 1. Introduction

Aging is an inevitable biological process characterized by a gradual decline in physiological and cognitive functions, which increases the risk of age-related brain diseases marked by oxidative stress, neuroinflammation, and neurodegeneration [1]. Additionally, the decline in motor function associated with aging can lead to a reduction in muscle mass, potentially influencing cognitive impairment through a decrease in myokines—hormones secreted by muscles during exercise [2].

Exercise has been shown to slow down age-related changes in brain structure and function [3,4]. Physical activity boosts the production of neurotrophic factors, promoting neuroplasticity, enhancing antioxidant capacity, and reducing neuroinflammation [5]. Previous studies have demonstrated that these physiological effects can enhance cognitive function by preventing neuronal damage and promoting cell regeneration during the aging process [6,7]. Exercise has been shown to confer neuroprotective benefits through several signaling pathways, notably involving myokines such as fibronectin type III domain-containing protein 5 (FNDC5), which is cleaved to release irisin. FNDC5/irisin plays a crucial role in enhancing synaptic plasticity and cognitive functions, particularly in Alzheimer’s disease models [8]. These exercise-induced myokines have been shown to play critical roles in promoting neuronal health and function [9]. A decline in these myokines with aging can lead to neuronal damage and reduce the restorative capacity of neurons [10].

Myokines play roles similar to insulin, exerting various physiological effects such as controlling blood sugar, increasing insulin sensitivity, and promoting fatty acid oxidation [11,12]. A reduction in myokines is associated with a decline in brain function, contributing to age-associated cognitive impairment [2,13]. Irisin, in particular, has various physiological functions, including regulating energy metabolism, improving insulin sensitivity, and possessing anti-inflammatory properties [14]. Aging is associated with a decline in brain function, potentially mediated by reduced irisin levels. FNDC5, upregulated in skeletal muscle during exercise, interacts with peroxisome proliferator-activated receptor gamma coactivator 1 alpha (PGC-1α), a key regulator of exercise-induced metabolic adaptation [15]. Exercise enhances PGC-1α expression in muscle and, through the PGC-1α/FNDC5 signaling pathway, promotes the production of BDNF in the brain, a neurotrophin crucial for cognitive functions such as learning and memory [16].

Nuclear factor erythroid 2–related factor 2 (Nrf2) is a transcription factor pivotal in cellular defense against oxidative stress and neuroinflammation [17]. This protein regulates the cellular oxidative stress response by activating various antioxidant and detoxifying enzymes through binding to DNA sequences known as antioxidant response elements (AREs) [18]. The activation of Nrf2 is implicated in a range of physiological and pathological processes, including anti-inflammatory responses, neuronal survival and protection, and the prevention and treatment of neurological and metabolic disorders. Nrf2 is activated by diverse stimuli, such as reactive oxygen species and neuroinflammation, subsequently translocating to the nucleus to induce the expression of neuroprotective genes [19]. Activation of Nrf2 has been shown to enhance cognitive function in aging and neurodegenerative diseases while mitigating neuronal cell death [20]. Conversely, Nrf2 deficiency is associated with an accelerated decline in cognitive function during aging.

Sirt1 is an NAD+-dependent histone deacetylase located primarily in the cell nucleus, contributing to cellular regulation and implicated in gene expression, metabolism, and delayed cellular aging [21]. Sirt1 promotes the expression of PGC-1α, a protein expressed in muscle cells, playing a role in regulating mitochondrial biogenesis and oxidative metabolism [22]. These interactions are important in preventing and treating age-related diseases.

The purpose of this study was to investigate whether an 8-week exercise intervention activates the FNDC5/irisin pathway and whether it exerts antioxidant and anti-inflammatory effects via Sirt1 and Nrf2 activation. Age-related cognitive impairment is often linked to increased oxidative stress and inflammation, which can damage neuronal function. By activating these pathways, exercise may improve neuronal resilience and function. Furthermore, we aimed to determine the role of exercise training in improving memory and ameliorating age-related cognitive impairment in older mice.

## 2. Materials and Methods

### 2.1. Animals

First, 9-week-old and 12-month-old C57BL/6J male mice were purchased from Koatech (Pyeongtaek, Gyeonggi-do, Republic of Korea) and were given 1 week to adapt. Old mice were raised until 17 months of age. Prior to exercise training, mice were randomly assigned to two groups: control and exercise training. The following groups were used: young control (Y, 9 weeks old, *n* = 6), aging control (A, 17 months old, *n* = 6), and aging + exercise training (A + Ex, 17 months old, *n* = 6). Animals were housed in a regulated environment with a temperature of 22 ± 2 °C, 60% humidity, and a controlled 12 h light–dark cycle. Food was provided ad libitum. This study followed the guidelines for the management and use of laboratory animals of the National Institutes of Health and was approved by the Institutional Animal Care and Use Committee of Kyung Hee University (KHSASP-22-466). The Y + Ex group was included in the behavioral experiments; however, results from this group were not presented in the final analysis, as no significant differences were observed compared to the young control (Y) group. This decision was made to focus on the aging-related effects and the impact of exercise on the aged mice.

### 2.2. Exercise Performance

**Figure 1:**
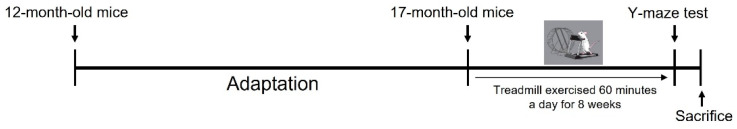
Schematic of the treadmill exercise protocol for 17-month-old mice.

The 17-month-old mice in the exercise group underwent a treadmill training regimen for 60 min per day, 5 days a week, over the course of 8 weeks. Prior to each session, mice were allowed 3 to 5 min to acclimate to the treadmill environment. The exercise protocol was structured in three phases: the first 10 min at a speed of 5 m/min on a 0° incline, the following 10 min at 8 m/min on a 0° incline, and the final 40 min at 10 m/min on a 0° incline. Mice were randomly assigned to the exercise training group before the start of the regimen to ensure unbiased allocation (Figure 1).

### 2.3. Behavioral Test (Y-Maze)

The Y-maze test is employed to evaluate spatial working memory and referential memory, both of which are components of short-term memory. The maze consists of three arms, labeled A, B, and C. Mice are placed in the starting arm, area C, and their spontaneous alternation behavior is recorded over a 6 min period. An independent researcher, blinded to the group assignments, recorded the entries to ensure unbiased data collection. An entry was counted when a mouse’s hind legs completely passed the midpoint of an arm. An alternation is defined as sequential entries into three different arms (e.g., ABC, BCA), without repetition. A higher alternation rate suggests superior spatial working memory. The formula used to calculate the spontaneous alternation rate is as follows:

The spontaneous alternation (%) = [(No. of alternations)/(Total arm entries − 2)] × 100.

### 2.4. Tissue Preparation

Upon completion of the experiment, animals were adequately sedated and anesthetized by a trained researcher following approved protocols and guidelines. Anesthesia was induced using isoflurane, delivered via inhalation through a vaporizer, ensuring that a sufficient level of anesthesia was achieved. Once confirmed, euthanasia was performed through exsanguination to ensure complete drainage of blood. Within each group—comprising young controls (Y, 9 weeks old, *n* = 6), aging controls (A, 17 months old, *n* = 6), and aging mice with exercise training (A + Ex, 17 months old, *n* = 6)—the animals were further divided for specific analyses. Three mice from each group were allocated for immunohistochemistry (IHC) or immunofluorescence (IF), while the remaining three were designated for Western blotting (WB). For the IHC and IF analyses, the brains were collected and initially fixed in a 4% paraformaldehyde (PFA) solution in 100 mM phosphate buffer (PB). Following initial fixation, the brains were post-fixed in the same PFA solution and then immersed in a 30% sucrose solution for three days to achieve dehydration. After this process, the brains were cryosectioned into 30-µm thick coronal sections using a microtome within a cryostat maintained at −23 °C. For Western blotting, the brains were rapidly frozen and stored at −80 °C to preserve protein integrity for subsequent analysis.

### 2.5. Immunohistochemistry and Immunofluorescence

Brain sections were processed using the free-floating method to visualize target proteins via immunohistochemistry and immunofluorescence. Initially, endogenous peroxidase activity was blocked by incubating the sections in 3% H_2_O_2_ in PBS for 20 min at room temperature. The tissues were then blocked with a solution containing 1% bovine serum albumin (BSA) and 10% normal goat serum in PBS for 2 h. Following blocking, the brain tissues were incubated overnight at 4 °C with primary antibodies against irisin (1:1000; Novus Biologicals, Littleton, CO, USA) and calbindin (1:1000; Abcam, Cambridge, UK). The next day, sections were incubated with an anti-rabbit secondary antibody (1:200, Vector Laboratories, Burlingame, CA, USA) for 2 h at room temperature. Bound secondary antibodies were amplified using an antibody-biotin-avidin-peroxidase complex solution (Vector Elite ABC Kit^®^, Vector Laboratories, Burlingame, CA, USA) for 1 h at room temperature, and visualization was achieved using 3,3′-diaminobenzidine tetrahydrochloride (DAB Kit, Vector Laboratories) for 5 min.

For immunofluorescence, sections were washed with 0.05 M PBS and incubated with a blocking solution consisting of 2% BSA and 10% normal rabbit and goat serum in 0.05 M PBS for 2 h at room temperature. Sections were then incubated overnight at 4 °C with antibodies targeting NeuN (1:1000; Abcam), Iba-1 (1:500; Abcam), anti-glial fibrillary acidic protein (GFAP; 1:1000; Abcam), Nrf2 (1:1000; Abcam), and Keap1 (1:500; Abcam). After PBS washing, sections were incubated with a mixture of Alexa Fluor 488-conjugated donkey anti-rabbit IgG (1:1000; Molecular Probes, Eugene, OR, USA) and Alexa Fluor 594-conjugated goat anti-mouse or goat IgG (1:1000; Molecular Probes) for 1 h at room temperature. Visualization was performed using a confocal microscope (Zeiss LSM 700, Oberkochen, Germany). Quantitative analysis of immunohistochemistry and immunofluorescence data was conducted using Image-Pro^®^ Plus software, version 6.0 (Media Cybernetics, Silver Spring, MD, USA) and ImageJ software version 1.53t (National Institutes of Health, Bethesda, MD, USA).

### 2.6. Fluoro-Jade C Staining

Fluoro-Jade C (FJC) staining was employed to identify degenerating neurons using the FJC staining kit (Biosensis, Thebarton, South Australia) according to the manufacturer’s instructions. This method specifically targets neurons undergoing degeneration. Quantitative analysis was conducted to determine the number of FJC-positive neurons in the hippocampal CA1 and cortex regions for each experimental group. The average number of FJC-positive neurons was graphically represented and analyzed using ImageJ software (National Institutes of Health, Bethesda, MD, USA).

### 2.7. Western Blotting

Proteins were extracted from the hippocampus using lysis buffer (RIPA, Thermo Fisher Scientific, Waltham, MA, USA), and their concentrations were determined using a colorimetric protein assay kit (Bio-Rad, Hercules, CA, USA). Subsequently, 20 µg of proteins were separated on a 10% SDS-polyacrylamide gel and transferred to a polyvinylidene fluoride (PVDF) membrane. The membrane was blocked with 5% skim milk in Tris-buffered saline with 0.1% Tween 20 (TBST; 10 mM Tris, pH 7.6, 150 mM NaCl, 0.1% Tween 20) for 1 h at room temperature (RT). Following blocking, the membrane was washed with TBST and incubated overnight at 4 °C with primary antibodies targeting FNDC5 (1:1000; Abcam, Cambridge, UK), PGC-1α (1:1000; Abcam, Cambridge, UK), IGF-1 (1:1000; Abcam, Cambridge, UK), IGF-2 (1:500; Abcam, Cambridge, UK), BDNF (1:2000; Santa Cruz, CA, USA), Sirt1 (1:500; Abcam, Cambridge, UK), CREB (1:2000; Cell Signaling, Danvers, MA, USA), pCREB (1:1000; Cell Signaling, Danvers, MA, USA), AKT (1:1000; Cell Signaling), pAKT (1:1000; Cell Signaling, Danvers, MA, USA), Nrf2 (1:1000; Abcam, Cambridge, UK), SOD1 (1:1000; Santa Cruz, CA, USA), GSTO1/2 (1:1000; Santa Cruz, CA, USA), and β-actin (1:10,000; Santa Cruz, CA, USA). After further TBST washing, the membrane was incubated with horseradish peroxidase-conjugated secondary antibodies (1:2000; Molecular Probes, OR, USA) for 1 h at RT. Band detection was performed using imaging systems (ChemiDoc, Bio-Rad) with a chemiluminescence Western blotting detection system (Clarity™ Western ECL Substrate, Bio-Rad) for visualization. Relative protein expression was analyzed using Image-Pro^®^ Plus software (Media Cybernetics, Silver Spring, MD, USA) and ImageJ software (National Institutes of Health, Bethesda, MD, USA).

### 2.8. Statistical Analysis

All data are presented as the mean ± standard error of the mean (S.E.M). Statistical analysis was conducted using SPSS software version 26.0 (IBM Corp., Chicago, IL, USA). Differences between groups were assessed using one-way ANOVA followed by Tukey’s post hoc test for multiple comparisons. *p*-values less than 0.05 were considered statistically significant.

## 3. Results

### 3.1. Exercise Alleviates Aging-Induced Cognitive Impiarment

**Figure 2:**
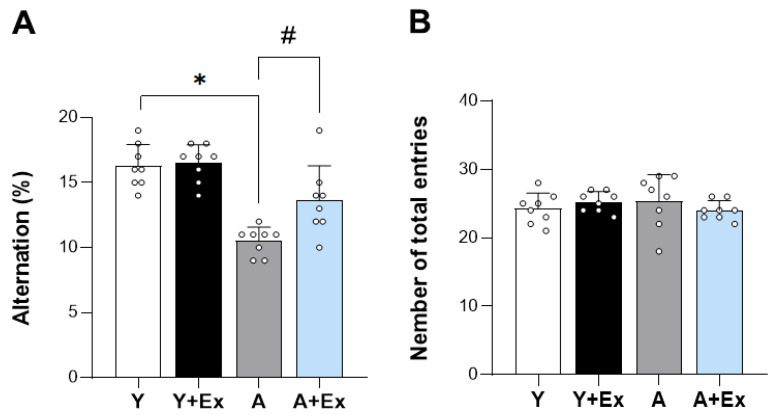
Effect of exercise on aging-induced cognitive impairment using the Y-maze test. This figure shows the impact of exercise on cognitive performance in aged mice, assessed using the Y-maze test. (**A**) The spontaneous alternation percentage (%) was significantly improved in aged mice that underwent exercise compared to aged mice without exercise, indicating enhanced cognitive function. (**B**) There were no significant differences in the total number of entries among all groups, suggesting that motor function remained unchanged. Data are presented as the mean ± standard deviation (SD), reflecting the biological variability within each group. * indicates *p* < 0.05 compared with the young group; # indicates *p* < 0.05 compared with the aging group. The following groups were used in the study: Young Control (Y, 9 weeks old, *n* = 6), Young + Exercise Training (Y + Ex, 9 weeks old, *n* = 6), Aging Control (A, 17 months old, *n* = 6), and Aging + Exercise Training (A + Ex, 17 months old, *n* = 6).

To assess the impact of exercise on age-related cognitive impairment, we conducted a Y-maze behavioral test. The results showed a significant decrease in alternation percentage (%), which is a measure of cognitive function, in the aged group compared to the young group (F = 28.082, *p* < 0.001). In contrast, the aged + exercise group exhibited a significant increase in alternation percentage, suggesting that exercise has the potential to alleviate age-related cognitive impairment (*p* < 0.001). These findings strongly indicate that exercise intervention can ameliorate cognitive impairment associated with aging (Figure 2A). Furthermore, upon examining the total entries, which serve as an indicator of motor function during the Y-maze test, we observed no significant differences in motor function among all groups (Figure 2B). This suggests that the observed cognitive improvements were not confounded by changes in motor ability.

### 3.2. Exercise Inhibits Aging-Induced Neuronal Death in the Hippocampus and Cortex

**Figure 3:**
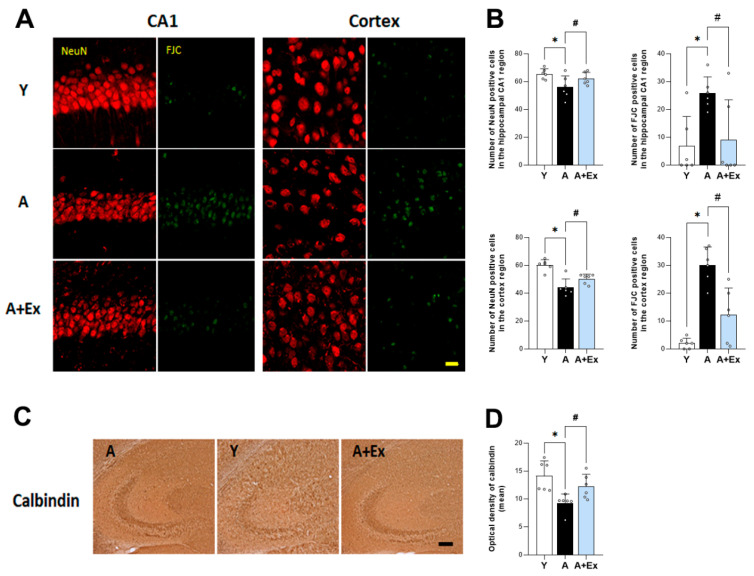
Effect of exercise on aging-induced neuronal death in the hippocampus and cortex regions. (**A**) Representative images of immunostaining for NeuN-positive cells and Fluoro-Jade C (FJC) in the hippocampal CA1 and cortex regions, illustrating the effects of aging. (**B**) Aged mice demonstrated a reduced number of neurons and an increased number of degenerative neurons in these regions. However, exercise in aged mice ameliorated neuronal death in both the hippocampal CA1 and cortex regions. (**C**) Representative images of immunostaining show changes in calbindin expression due to aging and exercise intervention. (**D**) Aged mice demonstrated reduced calbindin expression in the hippocampal CA3 region. However, exercise in aged mice improved calbindin expression in the hippocampal CA3 region. Data are presented as the mean ± standard deviation (S.D * indicates *p* < 0.05 compared with the young group; # indicates *p* < 0.05 compared with the aging group. Scale bar represents 50 μm (**A**), 200 μm (**C**). The following groups were used: young control (Y, 9 weeks old, *n* = 3), aging control (A, 17 months old, *n* = 3), and aging + exercise training (A + Ex, 17 months old, *n* = 3).

In aged mice, there was a significant decrease in neuronal cell markers, including NeuN, in both the hippocampal CA1 region (F = 9.121, *p* < 0.001) and the cerebral cortex (F = 39.320, *p* < 0.001) compared to young mice. However, exercise intervention significantly alleviated neuronal damage in the hippocampal CA1 region (*p* < 0.05) and cerebral cortex (*p* < 0.01) caused by aging (Figure 3A). Additionally, the expression of Fluoro-Jade C (FJC), a marker of dead neurons, was significantly increased in the hippocampal CA1 region (F = 5.464, *p* < 0.05) and cerebral cortex (F = 26.865, *p* < 0.001) in the aging group. Exercise intervention, however, reduced FJC expression in both the hippocampal CA1 region (*p* < 0.05) and cerebral cortex (*p* < 0.001), indicating a protective effect against neuronal death (Figure 3A). Our results demonstrate that exercise has a beneficial effect in preventing aging-induced neuronal death in the hippocampal CA1 and cortex regions. Furthermore, as shown in Figure 3B, the expression of irisin (F = 9.861, *p* < 0.001) and calbindin (F = 16.129, *p* < 0.001) in the hippocampal CA3 region decreased due to aging but increased following exercise intervention, highlighting the neuroprotective role of exercise.

### 3.3. Exercise Alleviates Activation of Microglia and Astrocyte in the Hippocampus

**Figure 4:**
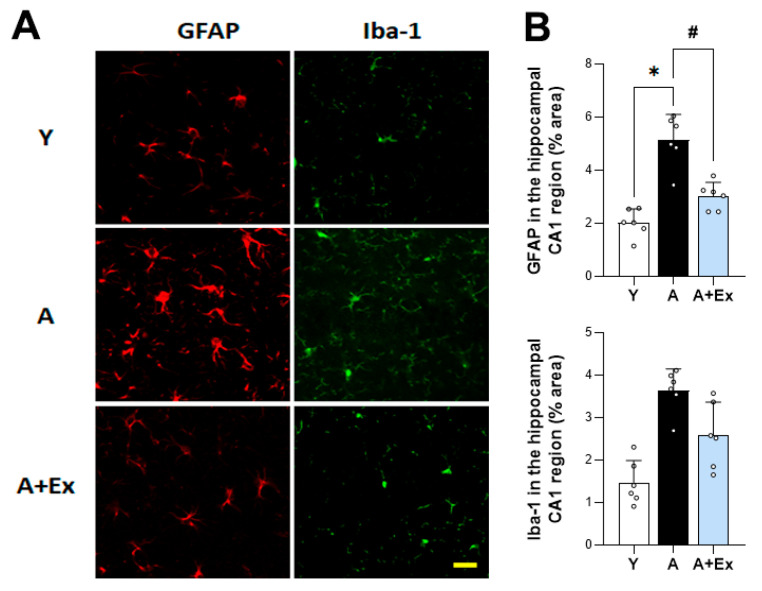
Effect of exercise on aging-induced microglia and astrocyte activation in the hippocampal CA1 region. (**A**) Representative images of immunostaining for GFAP and Iba-1 expression in the hippocampal CA1 region, illustrating changes in microglia and astrocyte activation. (**B**) Aged mice exhibited increased activation of microglia and astrocytes. However, exercise in aged mice reduced this activation in the hippocampal CA1 region. Data are presented as the mean ± standard deviation (S.D.). * indicates *p* < 0.05 compared with the young group; # indicates *p* < 0.05 compared with the aging group. Scale bar represents 50 μm. The following groups were used: young control (Y, 9 weeks old, *n* = 3), aging control (A, 17 months old, *n* = 3), and aging + exercise training (A + Ex, 17 months old, *n* = 3).

To evaluate the impact of aging and exercise on glial cell activation in the brain, we assessed the expression of glial fibrillary acidic protein (GFAP) and ionized calcium-binding adapter molecule 1 (Iba-1) in the hippocampus. In aged mice, there was a significant increase in the expression of GFAP (F = 31.097, *p* < 0.001) and Iba-1 (F = 18.714, *p* < 0.001) compared to young mice, indicating heightened activation of astrocytes and microglia, respectively. However, exercise intervention led to a significant decrease in the expression of both GFAP (*p* < 0.001) and Iba-1 (*p* < 0.05), suggesting that exercise can mitigate the excessive activation of microglia and astrocytes associated with aging (Figure 4). These findings imply that exercise not only alleviates cognitive impairment but also reduces neuroinflammatory processes linked to aging.

### 3.4. Effects of Treadmill Exercise on FNDC5/Irisin Expression in the Hippocampus and Gastrocnemius Muscle

**Figure 5:**
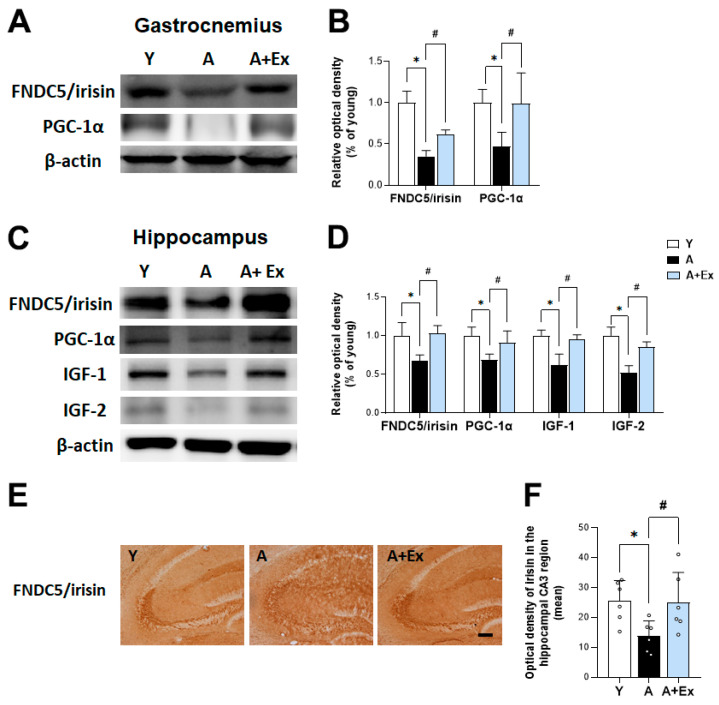
Effects of exercise on FNDC5/irisin and PGC-1α expression in the hippocampus and gastrocnemius. (**A**,**C**) Representative bands showing the protein expression of FNDC5/irisin, PGC-1α, IGF-1, and IGF-2 in the hippocampus and gastrocnemius muscle. (**B**,**D**) Aging resulted in decreased expression of FNDC5, PGC-1α, IGF-1, and IGF-2 in both the hippocampus and gastrocnemius compared to young mice. However, exercise intervention significantly improved the expression of these proteins in the hippocampus. Similarly, exercise increased the expression of FNDC5/irisin and PGC-1α in the gastrocnemius muscle. (**E**,**F**) Representative images of immunostaining illustrate changes in FNDC5/irisin expression due to aging and exercise intervention. Data are presented as the mean ± standard deviation (S.D.). * indicates *p* < 0.05 compared with the young group; # indicates *p* < 0.05 compared with the aging group. The scale bar represents 200 μm. The following groups were used: young control (Y, 9 weeks old, *n* = 3), aging control (A, 17 months old, *n* = 3), and aging + exercise training (A + Ex, 17 months old, *n* = 3).

Since irisin is cleaved from FNDC5, the detection method used inherently includes its precursor, FNDC5. Therefore, we collectively refer to FNDC5/irisin levels when discussing the immunoassay, considering their similar molecular weights. In Figure 5A, In the gastrocnemius muscle, aging was associated with decreased expression of FNDC5/irisin (F = 26.875, *p* < 0.001) and PGC-1α (F = 11.252, *p* < 0.001). However, exercise intervention led to increased expression of FNDC5/irisin (*p* < 0.05) and PGC-1α (*p* < 0.01). In Figure 5C, aging was correlated with reduced expression of FNDC5/irisin (F = 3.893, *p* < 0.05), PGC-1α (F = 7.781, *p* < 0.001), IGF-1 (F = 9.632, *p* < 0.01), and IGF-2 (F = 12.051, *p* < 0.001) in the hippocampus. Conversely, exercise intervention resulted in increased expression of FNDC5/irisin (*p* < 0.05), PGC-1α (*p* < 0.05), IGF-1 (*p* < 0.01), and IGF-2 (*p* < 0.05). The 8-week exercise regimen in aged mice elevated the expression of FNDC5/irisin-related pathway factors in both the brain and muscle, yielding beneficial effects on aging. In Figure 5E, the expression of FNDC5/irisin in the hippocampal CA3 region decreased due to aging but increased following exercise intervention (F = 9.861, *p* < 0.001).

### 3.5. Exercise Improves Aging-Induced BDNF/Sirt1/CREB/AKT Signaling Pathway in the Hippocampus

**Figure 6:**
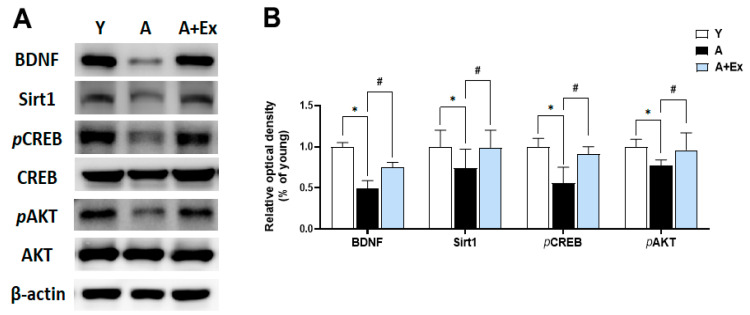
Effects of treadmill exercise on BDNF/Sirt1/CREB/AKT signaling pathway in the hippocampus. (**A**) Representative bands show the protein expression of BDNF, Sirt1, *p*CREB, and *p*AKT in the hippocampus. (**B**) Aging resulted in decreased expression of these proteins compared to young mice. However, exercise intervention significantly improved the expression of BDNF, Sirt1, *p*CREB, and *p*AKT in the hippocampus. Data are presented as the mean ± standard deviation (S.D.). * indicates *p* < 0.05 compared with the young group; # indicates *p* < 0.05 compared with the aging group. The following groups were used: young control (Y, 9 weeks old, *n* = 3), aging control (A, 17 months old, *n* = 3), and aging + exercise training (A + Ex, 17 months old, *n* = 3).

Brain-Derived Neurotrophic Factor (BDNF) plays a crucial role in promoting neuron survival and growth and is essential for learning and memory formation [23]. Sirtuin 1 (Sirt1) is pivotal in regulating cell survival and maintaining metabolic balance [24]. The cAMP response element-binding protein (CREB) and protein kinase B (AKT) signaling pathways are instrumental in regulating gene expression in the brain and are vital for brain survival and function. As shown in Figure 6, the expression of BDNF (F = 29.991, *p* < 0.001), Sirt1 (F = 24.968, *p* < 0.001), phosphorylated CREB (pCREB) (F = 6.014, *p* < 0.05), and phosphorylated AKT (*p*AKT) (F = 12.351, *p* < 0.01) in the hippocampus decreased with aging. However, exercise intervention significantly increased the expression of BDNF (*p* < 0.01), Sirt1 (*p* < 0.01), *p*CREB (*p* < 0.05), and *p*AKT (*p* < 0.01). These findings suggest that exercise can mitigate the decline in brain function due to aging by enhancing the BDNF, Sirt1, *p*CREB, and *p*AKT signaling pathways, which are crucial for maintaining brain function.

### 3.6. Exercise Upregulates Nrf2 Expression in the Motor Cortex and Hippocampus

**Figure 7:**
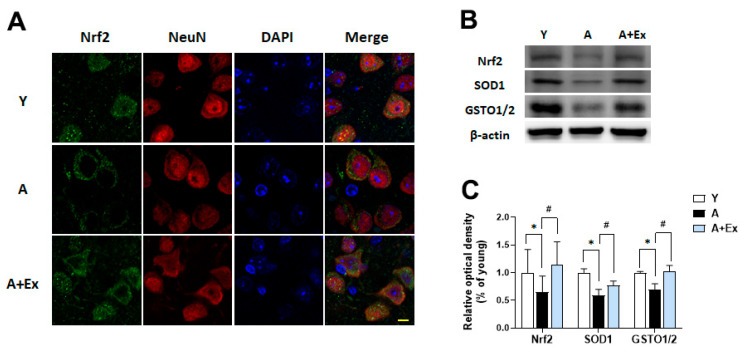
Effect of exercise on aging-induced Nrf2 dysfunction in the motor cortex and hippocampus. (**A**) Representative photographs of immunofluorescence staining show Nrf2 (green) and NeuN (red)-positive cells in the motor cortex. (**B**) Representative bands illustrate the protein expression of Nrf2, SOD1, and GSTO1/2 in the hippocampus. (**C**) Aging resulted in decreased expression of these proteins compared to young mice. However, exercise intervention significantly improved the expression of Nrf2, SOD1, and GSTO1/2 in the hippocampus. Data are presented as the mean ± standard deviation (S.D.). * indicates *p* < 0.05 compared with the young group; # indicates *p* < 0.05 compared with the aging group. The scale bar represents 20 μm. The following groups were used: young control (Y, 9 weeks old, *n* = 3), aging control (A, 17 months old, *n* = 3), and aging + exercise training (A + Ex, 17 months old, *n* = 3).

Nuclear factor erythroid 2-related factor 2 (Nrf2) acts as a transcription factor that regulates the antioxidant response, playing a crucial role in preventing age-related neuronal damage [20,25]. Aging is associated with decreased Nrf2 expression and increased oxidative stress in the brain. In the aging group, Nrf2 expression was primarily localized to the cytosol and was reduced, with no significant presence in the nucleus. In contrast, the exercise intervention group showed increased Nrf2 expression, with the protein evenly distributed between the nucleus and cytosol (Figure 7A). Additionally, the expression of Nrf2 (F = 5.137, *p* < 0.05), superoxide dismutase 1 (SOD1) (F = 13.054, *p* < 0.001), and glutathione S-transferase omega 1/2 (GSTO1/2) (F = 9.251, *p* < 0.01) in the brain decreased with aging. Exercise intervention led to increased expression of Nrf2 (*p* < 0.05), SOD1 (*p* < 0.05), and GSTO1/2 (*p* < 0.01) (Figure 7B,C). These findings suggest that aging heightens oxidative stress, which diminishes the levels of antioxidant-related proteins, while exercise can enhance these antioxidant factors, potentially mitigating age-related neuronal damage.

## 4. Discussion

A decline in cognitive function is a natural aspect of aging and can result from various factors, including alterations in brain structure and function, neuroinflammation, oxidative stress, and diminished blood flow [1]. Several animal studies have demonstrated that aged mice exhibit reduced learning and memory function compared to younger mice in the Morris water maze and Y-maze tests [26,27]. The Y-maze test, which is relatively simple and can be performed quickly, is useful for evaluating spatial memory in mice [28]. Consistent with previous studies, our study showed that aged mice exhibited cognitive impairment compared to young mice in the Y-maze test. However, when aged mice underwent treadmill exercise for 8 weeks, the spontaneous alternation rate in the Y-maze test increased. Based on these results, exercise has the potential to ameliorate cognitive impairment induced by aging.

Calbindin is a 28 kDa calcium-binding protein expressed in neurons, playing a crucial role in protecting neurons from excitotoxic damage, which can occur due to excessive levels of intracellular calcium [29]. The expression of calbindin in the hippocampus decreases with aging, and the depletion of calbindin exacerbates neuronal loss, apoptosis, mitochondrial dysfunction, and synapse loss. This reduction in calbindin is significant in the pathogenesis of Alzheimer’s disease [30]. The age-related reduction in calbindin in the hippocampus may contribute to neuronal vulnerability and cognitive impairment [31]. Irisin plays a protective role in the central nervous system, contributing to neurogenesis, reducing inflammation, alleviating oxidative stress, and restoring the balance of neurotrophic factors [32]. Exercise was found to improve the expression of calbindin, which had been reduced due to aging. This exercise intervention alleviated neurological vulnerability and cognitive impairment by mitigating the age-related reduction in calbindin in the hippocampal CA3 region. Additionally, our study included analyses of NeuN, a marker for mature neurons, and Fluoro-Jade C (FJC), a marker for degenerating neurons. We observed that the expression of NeuN was decreased in the hippocampus of aged mice, indicating reduced neuronal integrity. In contrast, FJC staining was increased, signifying heightened neuronal degeneration. However, exercise intervention resulted in increased NeuN expression and decreased FJC staining, suggesting that exercise not only enhances neuronal survival but also reduces neuronal degeneration in the aging hippocampus.

Normal microglia and astrocytes are responsible for synapse formation, elimination, and maintaining homeostasis [33]. Aging increases cytokine secretion through microglial activation in the brain [34]. This aging-induced hyperactivity of microglia and astrocytes can result in an increased inflammatory response, leading to neuronal damage in the brain and subsequent cognitive impairment [35]. Our results showed that the expression of GFAP and Iba-1 in the CA1 region of the hippocampus was higher in aged mice compared to young mice. However, exercise intervention reduced the expression of GFAP and Iba-1 in the hippocampus. These results confirm that exercise can protect against neural damage in the brain by reducing the activity of microglia and astrocytes, which is increased due to aging.

Exercise enhances the expression of peroxisome proliferator-activated receptor gamma coactivator 1 alpha (PGC-1α), leading to the generation of FNDC5/irisin [15]. FNDC5, which increases through exercise, is cleaved into irisin that is subsequently transported from muscles to the brain [36]. Our study demonstrated that the expression levels of FNDC5/irisin and PGC-1α were decreased in the hippocampus of aged mice but increased following exercise intervention. A previous study showed that 20-month-old female mice exhibited decreases in FNDC5/irisin and PGC-1α in the hippocampus, associated with aging-induced cognitive impairment [37]. However, it has been reported that FNDC5/irisin and PGC-1α levels increase along with cognitive function improvement through voluntary wheel running for 13 weeks. Additionally, swimming exercise has been reported to reduce apoptosis and neuroinflammation in the hippocampus by increasing PGC-1α levels in 20-month-old male Sprague-Dawley rats [38].

In our study, we observed that the levels of Sirt1, BDNF, pCREB, pAKT, PGC-1α, IGF-1, and IGF-2 were decreased due to aging. Sirt1 is a gene regulatory protein that influences cellular metabolism, aging, and inflammatory responses [24]. A reduction in Sirt1 during the aging process is known to contribute to cognitive impairment [39]. Sirt1 plays a crucial role in neuron survival and neurotransmission by regulating the expression of BDNF and PGC-1α in the brain [40]. Aging also reduces BDNF levels, impairing neuron survival, growth, and cognitive function [41]. CREB is a brain protein essential for enhancing memory and learning ability, while AKT is vital for supplying oxygen and nutrients to cells and regulating their survival, growth, and division [42,43]. Sirt1 is known to promote CREB activation and increase AKT and BDNF levels. Therefore, enhancing Sirt1 levels can be significant in preventing or treating cognitive impairment due to aging [44]. Exercise has been shown to increase Sirt1 and BDNF levels, positively impacting aging-induced cognitive impairment and improving brain function. IGF-1 in the central nervous system (CNS) is an important anabolic hormone that decreases with age [45]. IGF-1 and IGF-2 are insulin-like growth factors that promote cell proliferation, differentiation, and survival, exerting insulin-like metabolic effects in most cell types and tissues [46]. IGF-1 promotes anti-aging effects, cell regeneration, healthy metabolism, improved brain function, decreased inflammation, and enhanced immune function [47]. The expression of IGF-2 was significantly upregulated in the hippocampus following moderate treadmill exercise. Consistent with our results, 8 weeks of treadmill exercise in 18-month-old male mice was associated with improved cognitive function by increasing levels of FNDC5/irisin, PGC-1α, IGF-1, and IGF-2 in the hippocampus.

Age-related cognitive impairment is influenced by various factors, with this study focusing on oxidative stress, which escalates with aging [1]. Oxidative stress can lead to DNA damage, protein abnormalities, and cell membrane damage, resulting in the degradation of cell function and cell death [48]. Nrf2 is a key regulator that recognizes and activates oxidative stress signals within cells, inducing an antioxidant response to protect neurons [18]. Once activated, Nrf2 translocates to the nucleus and promotes the expression of various genes that enhance antioxidant responses [49]. However, a decrease in Nrf2 due to aging leads to increased oxidative stress as the antioxidant function diminishes [50].

Our findings demonstrated that Nrf2 expression was diminished with aging and predominantly retained in the cytoplasm. In contrast, treadmill exercise markedly enhanced Nrf2 expression and promoted a shift toward nuclear localization, suggesting that exercise may facilitate Nrf2 nuclear translocation and thereby contribute to the restoration of antioxidant signaling in aged brains. Consistently, treadmill exercise activated the Nrf2–ARE pathway, as evidenced by increased expression of downstream antioxidant enzymes such as SOD1 and GSTO1/2 in the hippocampus [51,52].

Recent evidence has demonstrated the multifaceted neuroprotective role of FNDC5/irisin, particularly in aging and neurodegenerative conditions. In addition to its well-established identity as an exercise-induced myokine, FNDC5 functions critically in maintaining cellular redox balance and mitochondrial integrity. Specifically, FNDC5 stabilizes NRF2, thereby enhancing antioxidant defenses and mitochondrial homeostasis, ultimately attenuating oxidative stress and mitochondrial dysfunction [53].

While our study provides insights into the contribution of oxidative stress to age-related cognitive impairment and the potential neuroprotective effects of exercise, several limitations should be noted. First, our mechanistic interpretation regarding the FNDC5/irisin–Nrf2 axis is associative rather than causal. We did not perform pharmacological or genetic interventions to establish Nrf2 dependence (e.g., Nrf2 inhibition with siRNA-mediated knockdown) nor irisin neutralization/reduction; thus, we avoided definitive causal claims and plan these experiments in follow-up work. Second, our functional assessments of antioxidant defense function were limited. While we report SOD1 activity (Figure S1), we did not conduct a broader enzyme activity panel nor direct measurements of reactive oxygen species (ROS) and redox status; incorporating assays for catalase, GPx, GSH/GSSG ratios, and ROS will provide a more comprehensive evaluation of oxidative stress reduction. Finally, further interrogation of FNDC5/irisin’s role in stabilizing Nrf2 and its impact on mitochondrial function (e.g., mitochondrial membrane potential, respiratory capacity, and biogenesis markers) may clarify additional mechanisms underlying its neuroprotective effects.

The present study further demonstrates that exercise-induced FNDC5/irisin signaling significantly improves cognitive function by reducing age-associated oxidative stress, neuronal damage, and glial activation. These beneficial effects are achieved through the activation of multiple cellular pathways modulated by myokines released during exercise, which exhibit both antioxidant and neurotrophic properties. The FNDC5/irisin pathway plays a pivotal role in this process. Irisin, produced in response to physical activity, crosses the blood–brain barrier and exerts neuroprotective effects by activating the Nrf2-ARE signaling pathway. This pathway enhances the brain’s antioxidant capacity, reducing oxidative stress—a major contributor to neuronal damage and cognitive decline in aging. Furthermore, exercise stimulates the PGC-1α/FNDC5/BDNF axis, promoting the expression of BDNF, essential for synaptic plasticity and neuronal survival. Collectively, these findings provide mechanistic insight into the cognitive benefits of exercise and suggest that targeting the FNDC5–Nrf2 signaling axis through physical activity may represent a promising non-pharmacological strategy to preserve cognitive function and enhance quality of life in aging populations.

## Data Availability

The original contributions presented in this study are included in the article and Supplementary Material. Further inquiries can be directed to the corresponding author.

## Supplementary Materials

The following supporting information can be downloaded at: https://www.mdpi.com/article/10.3390/antiox14101239/s1, Figure S1. Changes in SOD Activity in Experimental Groups. Figure S2. Body Weight Changes Before and After Exercise Training (ET).
