# Supplementary material for: Exercise-Induced FNDC5/Irisin Ameliorates Cognitive Impairment in Aged Mice, Associated with Antioxidant and Neurotrophic Responses
Source: Antioxidants (Basel). 2025 Oct 15;14(10):1239. doi: 10.3390/antiox14101239 (PMC12561080; doi:10.3390/antiox14101239)
Supplement: Supplementary file 1 [file antioxidants-14-01239-s001.zip › antioxidants-3741434-supplementary.pptx]

## Slide 1
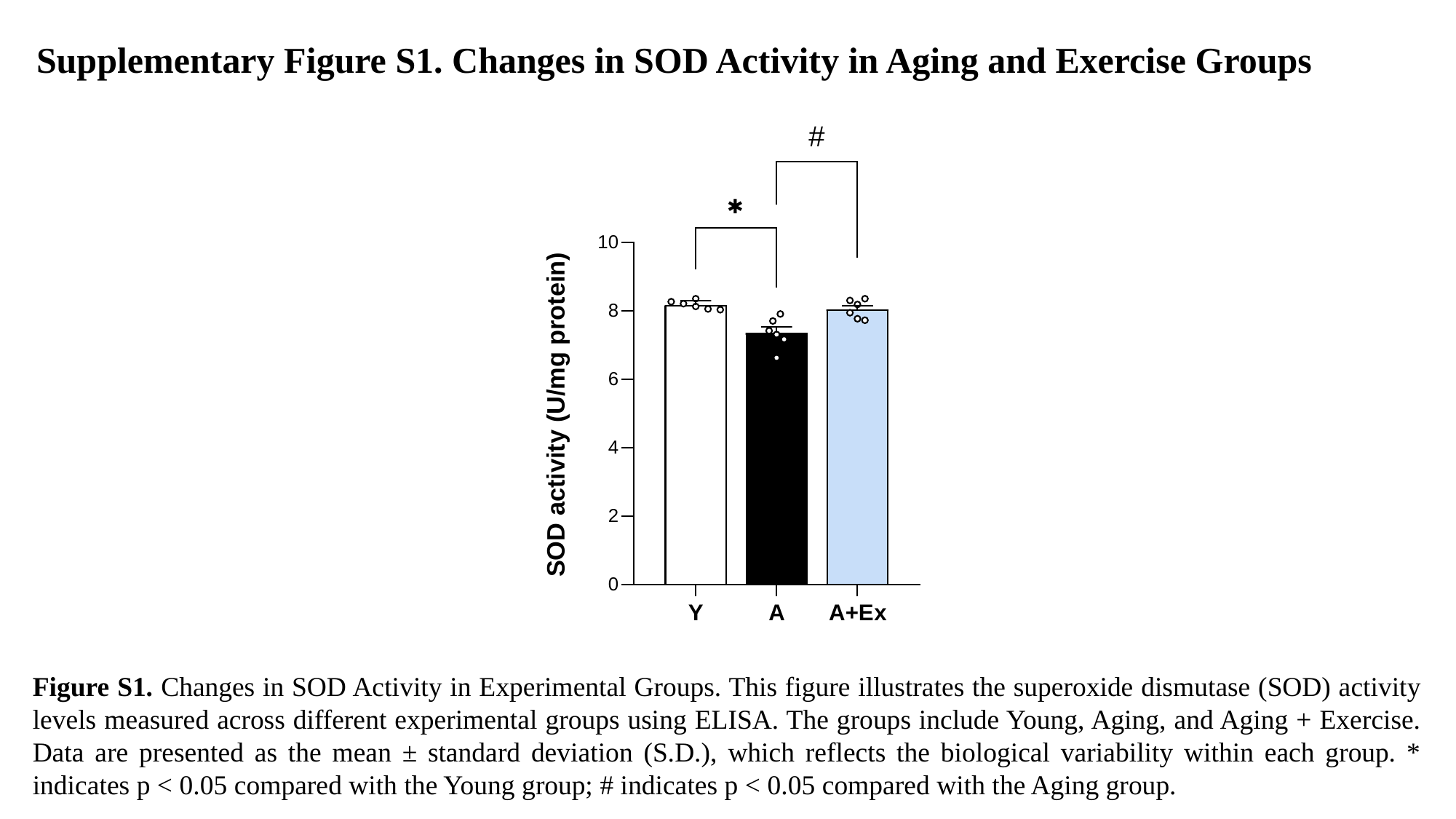

Supplementary Figure S1. Changes in SOD Activity in Aging and Exercise Groups
Figure S1. Changes in SOD Activity in Experimental Groups. This figure illustrates the superoxide dismutase (SOD) activity levels measured across different experimental groups using ELISA. The groups include Young, Aging, and Aging + Exercise. Data are presented as the mean ± standard deviation (S.D.), which reflects the biological variability within each group. * indicates p < 0.05 compared with the Young group; # indicates p < 0.05 compared with the Aging group.

## Slide 2
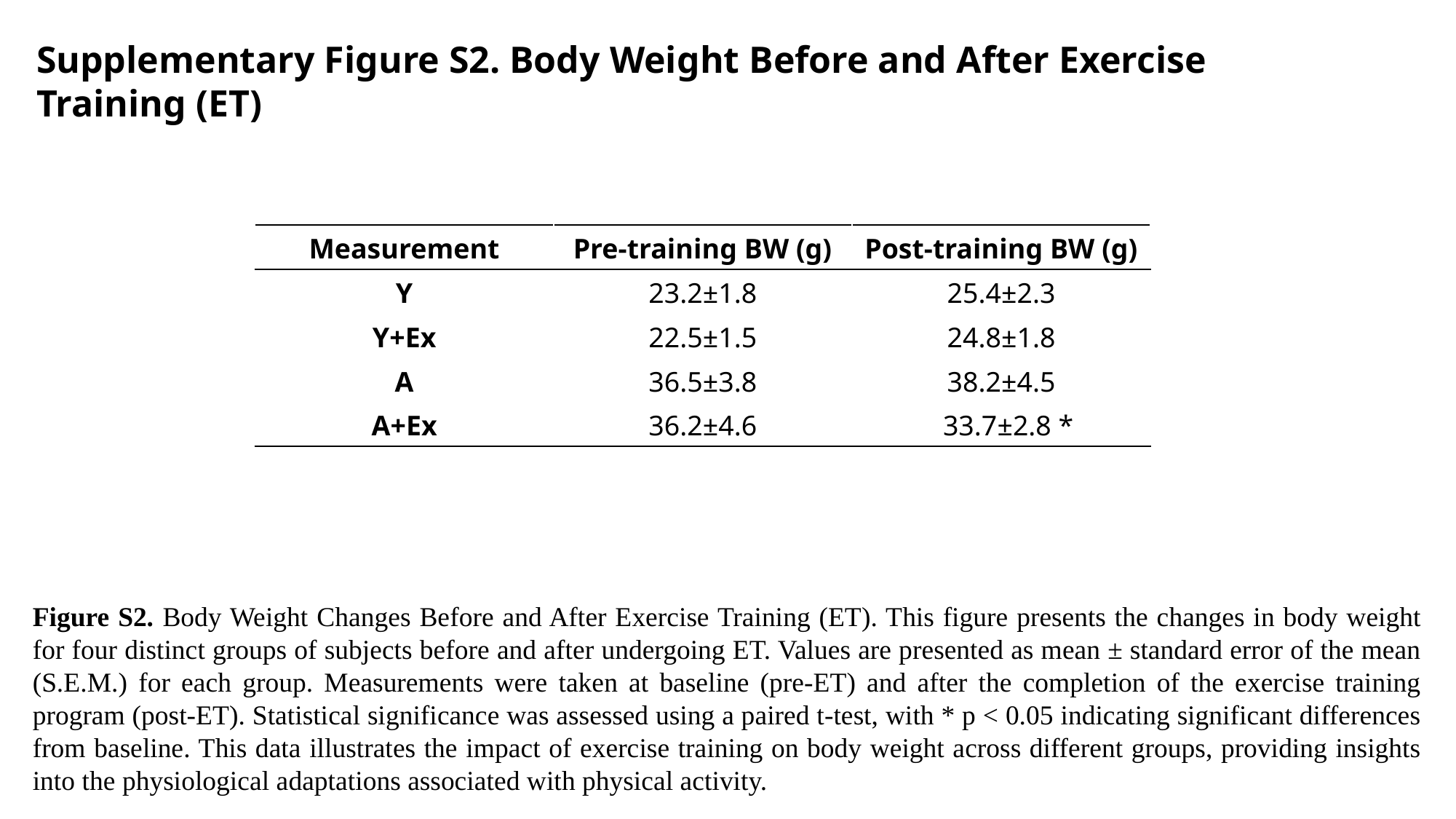

Supplementary Figure S2. Body Weight Before and After Exercise Training (ET)
| Measurement | Pre-training BW (g) | Post-training BW (g) |
| --- | --- | --- |
| Y | 23.2±1.8 | 25.4±2.3 |
| Y+Ex | 22.5±1.5 | 24.8±1.8 |
| A | 36.5±3.8 | 38.2±4.5 |
| A+Ex | 36.2±4.6 | 33.7±2.8 \* |
Figure S2. Body Weight Changes Before and After Exercise Training (ET). This figure presents the changes in body weight for four distinct groups of subjects before and after undergoing ET. Values are presented as mean ± standard error of the mean (S.E.M.) for each group. Measurements were taken at baseline (pre-ET) and after the completion of the exercise training program (post-ET). Statistical significance was assessed using a paired t-test, with * p < 0.05 indicating significant differences from baseline. This data illustrates the impact of exercise training on body weight across different groups, providing insights into the physiological adaptations associated with physical activity.
